# Current Thoughts on Maternal Nutrition and Fetal Programming of the Metabolic Syndrome

**DOI:** 10.1155/2013/368461

**Published:** 2013-02-14

**Authors:** Bonnie Brenseke, M. Renee Prater, Javiera Bahamonde, J. Claudio Gutierrez

**Affiliations:** ^1^Department of Biomedical Sciences and Pathobiology, Virginia Tech, Blacksburg, VA 24061, USA; ^2^Department of Pathology, Campbell University School of Osteopathic Medicine, Buies Creek, NC 27506, USA; ^3^Department of Biomedical Sciences, Edward Via College of Osteopathic Medicine, 2265 Kraft Drive, Blacksburg, VA 24060, USA

## Abstract

Chronic diseases such as type 2 diabetes and cardiovascular disease are the leading cause of death and disability worldwide. Although the metabolic syndrome has been defined in various ways, the ultimate importance of recognizing this combination of disorders is that it helps identify individuals at high risk for both type 2 diabetes and cardiovascular disease. Evidence from observational and experimental studies links adverse exposures in early life, particularly relating to nutrition, to chronic disease susceptibility in adulthood. Such studies provide the foundation and framework for the relatively new field of developmental origins of health and disease (DOHaD). Although great strides have been made in identifying the putative concepts and mechanisms relating specific exposures in early life to the risk of developing chronic diseases in adulthood, a complete picture remains obscure. To date, the main focus of the field has been on perinatal *undernutrition* and specific nutrient *deficiencies;* however, the current global health crisis of overweight and obesity demands that perinatal *overnutrition* and specific nutrient *excesses* be examined. This paper assembles current thoughts on the concepts and mechanisms behind the DOHaD as they relate to maternal nutrition, and highlights specific contributions made by macro- and micronutrients.

## 1. Introduction

The metabolic syndrome has become a major public health challenge with an estimated 22% of US adults having this condition [1]. A consensus group for the International Diabetes Federation defines metabolic syndrome as central obesity, plus any two of the following: raised triglycerides, reduced high-density lipoprotein (HDL) cholesterol, raised fasting plasma glucose, and raised blood pressure [2]. The consensus group also recommends additional criteria that should be part of further research into the metabolic syndrome including tomographic assessment of visceral adiposity and liver fat, biomarkers of adipose tissue (leptin, adiponectin), and glucose tolerance testing. The cause of the syndrome remains obscure but the pathophysiology seems to be largely attributable to insulin resistance, excessive flux of fatty acids, and a chronic proinflammatory state [3]. There is no specific treatment for metabolic syndrome. Therapeutics include lifestyle changes (e.g., weight reduction and increased physical activity) and pharmaceutical agents, but prevention would be preferred. A mounting body of evidence indicates that certain adverse exposures during the perinatal period contribute to the development of the metabolic syndrome. This early life stage may offer an attractive point in the disease process for prevention and intervention strategies. 

In the 1970s Forsdahl, using official statistical data on Norwegian counties, reported that poverty during adolescence, followed by prosperity, was positively correlated with risk of death from coronary heart disease [4]. Although no biological mechanisms were identified, Forsdahl speculated that some form of permanent damage caused by the nutritional deficit may be involved [4]. In 1986, Barker and colleagues began publishing reports on the association between an adverse intrauterine environment, as determined primarily by low birth weight, and an increased risk of coronary heart disease later in life. These studies involved examining men and women in middle and late life whose body measurements at birth were recorded in the archives and records offices of Britain [5, 6]. Upon further investigation, it was found that the correlation between low birth weight and heart disease was also present between low birth weight and type 2 diabetes with the prevalence of impaired glucose tolerance and type 2 diabetes falling progressively with increasing birth weight [7]. Moreover, in a follow-up study, an association between birth weight and the presence of metabolic syndrome was discovered [8]. In this study metabolic syndrome was defined as impaired glucose tolerance, hypertension, and elevated triglycerides. In both men and women, the prevalence of metabolic syndrome fell progressively with increasing birth weight. Of the 64-year-old men whose birth weights were 2.95 kg (6.5 pounds) or less, 22% had metabolic syndrome and their risk of developing the syndrome was more than 10 times greater than that of men whose birth weights were more than 4.31 kg (9.5 pounds) [8, 9]. The association between metabolic syndrome and low birth weight prompted the authors to suggest that the metabolic syndrome, also called syndrome X, be renamed “the small-baby syndrome” [8]. Collectively, these studies generated the Forsdahl-Barker hypothesis, recognizing Forsdahl as the original source of the idea and Barker as the developer of the concept [10]. In the decades following their initial publications, the Forsdahl-Barker hypothesis has become more well known as the “fetal origins” hypothesis and has produced a new branch of scientific knowledge and inquiry known as the developmental origins of health and disease (DOHaD). 

**Figure 1:**
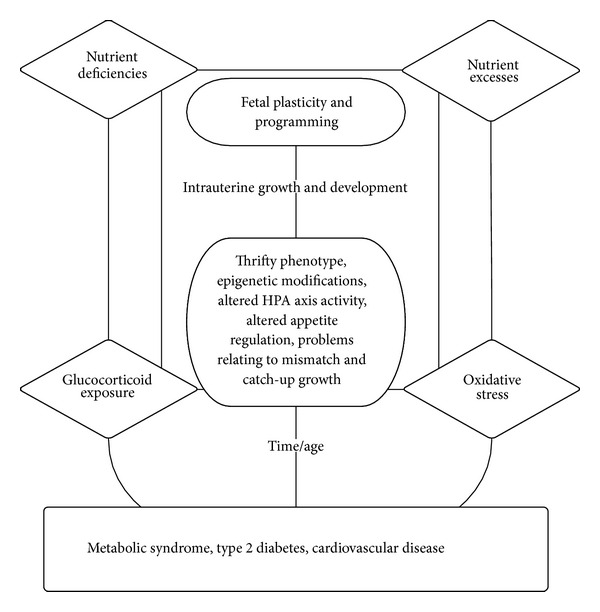
Schematic representation of the relationship between nutrient exposures and the concepts and mechanisms underlying the developmental origins of health and disease (DOHaD).

Historically, investigations on the fetal origins hypothesis have focused on maternal undernutrition and specific nutrient deficiencies. Today, the world faces the dual burden of malnutrition that encompasses both under- and overnutrition [11]. It is estimated that maternal and child undernutrition is the underlying cause of 3.5 million deaths globally [12]. While at the same time overweight and obesity are major public health challenges in both developed and developing regions of the world. In 2005, 33% of the world's adult population (1.3 billion people) was overweight or obese, and it is estimated that by 2030, up to 57.8% of the world's adult population (3.3 billion people) will be overweight or obese [13]. Adding to the complexity of the dual burden of malnutrition is the influence of socioeconomic status. By and large, in developed countries lower socioeconomic status is associated with both lower birth weights among the offspring of women and a higher prevalence of obesity among women [14]. As a result, failure to control for social position could obscure a relationship between high birth weight and subsequent obesity and obesity-related disorders [15]. Incidentally, although the vast majority of the literature concerning the DOHaD is focused on the relationship between small size at birth and increased incidence of disease in adult life, it is now recognized that higher incidences of disease occur in both those born small and those born large, thus reflecting a U-shaped curve [15, 16]. This paper provides a brief overview of the putative concepts and mechanisms behind the DOHaD and assembles thoughts on how alterations in maternal consumption of specific nutrients may irreversibly direct fetal programming of the metabolic syndrome (Figure 1). 

## 2. Concepts and Mechanisms 

### 2.1. Developmental Plasticity, Programming, and Mismatch

For most organs and systems the critical period of plasticity is during intrauterine development [17]. Developmental plasticity is the ability of an organism to change its phenotype in response to changes in the environment [18]. If this change or adaptation is permanent, it is considered a “programming” change and is associated with persistent effects in structure and/or function [18, 19]. Programming is an established biological phenomenon that is exemplified in nature. Gluckman and Hanson use the example of the meadow vole (*Microtus pennsylvanicus*) to illustrate programming [16]. Meadow moles born in autumn have a thicker hair coat than those born in the spring [20]. This occurs as a response of the fetus to maternally derived signals for day length [21]. A thick coat reflects an adaptive programming response to help ensure survival in the cold environment that was predicted while *in utero* [16]. Barker uses the functional activity of the sweat glands to illustrate plasticity and programming [17]. Humans have similar numbers of sweat glands at birth, but they are essentially nonfunctional. In the first few years of life a proportion of the glands become functional depending on the temperature to which the child is exposed. The hotter the conditions, the greater number of functional sweat glands. After a few years the process is complete and the number of functional sweat glands is fixed [17]. Programming changes can also be experimentally-induced as demonstrated by examination of adult (100-day-old) female rats that were given a single subcutaneous injection of testosterone at 2, 5, 10, or 20 days of age [22]. Those that were injected between birth and 10 days of age had lasting changes in organ weight, specifically larger adrenal and pituitary weights and smaller ovarian and uterine weights, as well as absent corpora lutea, failure to develop normal female sexual behavior, and permanent sterility [22, 23]. Theses aberrations in morphology and physiology were much reduced or absent in those rats injected at 10 or 20 days of age, indicating that the period of plasticity during which rats are androgen sensitive is between birth and 10 days of age [22]. 

In most cases programming is beneficial for the health and survival of the organism. However, the problem of “mismatch” occurs when individuals developmentally adapted to one environment are exposed to another [24]. Revisiting the functional activity of the sweat glands to provide an example, the child who has experienced hot conditions will be better adapted to such conditions in later life because more functioning sweat glands provide the ability to cool down faster. While the child who has experienced cool conditions faces the problem of mismatch and will be unable to cool down as easily in hot conditions because of fewer functioning sweat glands. Other examples of this mismatch phenomenon include people whose birth weights were towards the lower end of normal that subsequently grow up in affluent societies being at increased risk for hypertension, type 2 diabetes, and cardiovascular disease [16, 25]. The problem of mismatch is thought to be involved in the current “epidemic” of type 2 diabetes and cardiovascular disease in the young adult and middle-aged populations of India [26]. Several countries, including India, are undergoing swift economic and nutritional transitions, exposing individuals to conditions that promote weight gain [27]. The current surge in metabolic and cardiovascular disease in India may be being fueled by a combination of undernutrition in early life and overnutrition in later life. In a study of Indian children, those born small and where relatively fat and tall by 8 years of age had the most adverse risk profiles for cardiovascular disease, including components of the metabolic syndrome. The authors commented on the possible importance of preventing childhood obesity in the prevention of disease in the low birth weight members of the Indian population [26]. It is important to note that Indians as a race are more prone to the development of the metabolic syndrome, compared to Caucasians, as a result of having a phenotype of high fat mass and low lean mass [28].

### 2.2. Thrifty Phenotype Hypothesis

The “thrifty phenotype” hypothesis, put forth by Hales and Barker, proposes that poor fetal and early postnatal nutrition imposes mechanisms of nutritional thrift upon the growing individual. In conditions of severe intrauterine deprivation the developing fetus may lose functional and structural units such as pancreatic *β* cells, nephrons, and cardiomyocytes [29]. Such changes have been deemed an adaptive mechanism to ensure the survival of the fetus. Alternatively, the changes may reflect developmental malformations analogous to teratogenesis [16]. The thrifty phenotype hypothesis is based on a study of 468 men in which the percentage of those with impaired glucose tolerance or type 2 diabetes fell progressively with increasing birth weight and weight at 1 year [30]. From this research it was hypothesized that poor intrauterine nutrition results in the growth of vital organs, specifically the brain, at the expense of other organs (i.e., endocrine pancreas resulting in *β* cell hypoplasia). Such adaptations may increase the chance of fetal survival by means of “brain sparing” but result in difficulty in coping with nutritional abundance as an adult. 

Interestingly, the thrifty phenotype hypothesis has been challenged by the “fetal salvage” hypothesis which offers a different explanation for the insulin resistance seen in those affected by intrauterine growth restriction [31]. In this alternate explanation it is not hypoplasia of the pancreatic *β* cells that leads to impaired glucose tolerance, but rather it is that the fetus develops peripheral insulin resistance. It is this peripheral insulin resistance that ensures that adequate amounts of glucose are delivered to essential organs such as the brain with subsequent reduced delivery to nonessential tissues such as skeletal muscle. Support for the fetal salvage hypothesis comes from intrauterine growth restriction studies in rats. In these rodent models, glucose transport (glucose uptake and glucose transporter mRNA and protein levels) is reduced in lung and skeletal muscles of growth-restricted fetuses, whereas glucose transport is unaffected in brain [32, 33]. 

The kidney is another organ thought to be affected by nutritional thrift. In both human and animal studies small offspring, due to maternal undernutrition and other causes, have reduced numbers of nephrons which has been strongly linked to hypertension [16, 34, 35]. In the human, nephrogenesis is completed *in utero*, and no new nephrons are formed after birth; therefore a nephron deficit present at birth persists throughout life [29, 36]. In a study of human infants affected by intrauterine growth restriction, nephron number estimates were below the control group [35]. A similar study found a 20% reduction in nephron number in human neonates with low birth weights as compared to normal birth weight neonates. Additionally, researchers found a positive relationship between weight at birth and the number of glomeruli as well as a negative correlation between weight at birth and glomerular volume [37]. This reduction in nephrons may reflect an adaptation that has the immediate advantage of energy and resource conservation but no long-term advantages [16, 34]. 

### 2.3. Catch-Up Growth

Catch-up growth, also known as compensatory growth, is where children return to their genetic trajectory for size after a period of growth delay or arrest. It may occur at any stage of growth but is most commonly observed in the first 2 years of life [38]. Studies have found that catch-up growth often results in overcompensation, whereby the organism exceeds normal weight and often has excessive fat deposition. This rapid and excessive growth has been associated with the development of adult obesity, insulin resistance, metabolic syndrome, and type 2 diabetes [38–40].

 In the Avon Longitudinal Study of Pregnancy and Childhood (ALSPAC) birth cohort, children who showed catch-up growth, as calculated by specified gains in standard deviation scores, between 0 and 2 years of age, were heavier, taller, and fatter (body mass index, percentage body fat, and waist circumference) at 5 years of age than other children [38]. A study that evaluated glucose tolerance and plasma insulin concentrations in more than 1400 young adults (26 to 32 years old) who had grown up in the city of Delhi, India, found an association between thinness in infancy and the presence of impaired glucose tolerance or diabetes in young adulthood [41]. As a group, the young adults in the study who had impaired glucose tolerance or diabetes were overweight. They were not, however, overweight or obese in childhood. Instead, they were characterized by their high rate of gain in body mass after the age of 2 years [41]. 

Cianfarani and colleagues speculate that the tremendous effort to recover lost growth shortly after birth involves insulin and insulin-like growth factors (IGF). Infants affected by intrauterine growth restriction have low concentrations of insulin and IGF-1 at birth, and normalization of these parameters occurs in the postnatal period [42, 43]. It is thought that tissues chronically depleted of insulin and IGF-1 throughout fetal life and then suddenly exposed to increased concentrations of the two hormones shortly after birth may counteract the actions of insulin by developing insulin resistance. Thus, in this proposed scenario, insulin resistance is serving as a metabolic defense mechanism to protect the organism against hypoglycemia [39]. In addition to insulin resistance and type 2 diabetes, accelerated weight gain in early life has been associated with elevated blood pressure [44, 45] and coronary heart disease [46]. 

The implication of catch-up growth is that rapidly enhancing early childhood growth by a nutrient enriched diet may cause harm overtime and that encouraging slower growth rates may actually be beneficial. In a study that provides evidence of the benefits of steady (i.e., not accelerated by dietary means) growth, investigators measured fasting concentrations of 32-33 split proinsulin, a marker of insulin resistance, in adolescents born preterm who had participated in randomized intervention trials of neonatal nutrition [47]. Fasting 32-33 split proinsulin concentration was greater in those given a nutrient-enriched diet than those given the lower-nutrient diet and was associated with greater weight gain in the first 2 weeks of life [47]. 

### 2.4. Oxidative Stress

Excessive reactive oxygen species can cause modulation of gene expression and/or direct damage to cell membranes and other molecules at critical developmental windows. Many believe that oxidative stress is the primary link between adverse fetal growth and later elevated risks of the metabolic syndrome, type 2 diabetes, and other disorders [40]. Smoking, hypertension/preeclampsia, inflammation/infection, obesity, and malnutrition are common causes of preterm and/or low birth weight as well as known sources of oxidative stress. Malnutrition can directly lead to a pro-oxidative state by means of creating protein and micronutrient deficiencies. Proteins provide amino acids needed for antioxidant synthesis, such as glutathione and albumin, and many micronutrients themselves are antioxidants, such as vitamins A, C, and E [40, 48]. Pancreatic *β* cells are particularly sensitive to reactive oxygen species because they are low in enzymatic antioxidant defense equipment [49]. It has been demonstrated that oxidative stress can blunt insulin secretion [50]. With the susceptibility of pancreatic *β* cells to oxidative stress, it is believed that early and ongoing exposures to oxidative insults could result in the eventual manifestations of the metabolic syndrome and related disorders [40]. 

Elevated oxidative stress has been observed among human infants born small for gestational age (SGA) as compared to those appropriate for gestational age (AGA) and among preterm as compared to term infants. It is important to note that preterm (birth at <37 weeks of pregnancy) infants are generally of low birth weight but are not necessarily growth restricted or small for gestational age. A study using cord blood to compare the status of oxidative stress between SGA infants born to undernourished mothers and AGA infants born to healthy mothers found elevated oxidative stress, as determined by increased quantities of malondialdehyde (one of the major products of lipid peroxidation), reduced quantities of the antioxidant glutathione, and decreased activity of the antioxidants superoxide dismutase and catalase in the SGA infants as compared to the AGA controls [51]. 

A human study determined vitamin A, C, and E levels in the umbilical cord blood of term and preterm infants and the blood of their mothers taken at the time of delivery [52]. Maternal vitamin A and E levels were higher than cord values, yet there was a positive correlation between maternal and cord levels of these two vitamins. Contrary to vitamins A and E, cord vitamin C levels were higher than maternal levels, and no significant correlation between cord and maternal vitamin C levels was present. In comparing vitamin levels in term and preterm infants, term babies had significantly higher levels of vitamins A, C, and E. The authors concluded that for vitamins A and E, neonatal levels are dependent on maternal levels and that preterm babies have fewer lipid-soluble antioxidant vitamins in their serum compared to term infants thus may be more susceptible to oxidative stress [52]. 

Gestational diabetes, a common cause of macrosomia, is associated with oxidative stress which could have effects on the developing infant. A study evaluating oxidative and antioxidative status in pregnant diabetic (gestational diabetes or type 1 diabetes) women between 26 and 32 weeks gestation demonstrated increased oxidative stress in those who were diabetic [53]. Levels of malondialdehyde were significantly higher, and vitamin A and E levels were significantly lower in all diabetic women than controls. Moreover, glutathione peroxidase and superoxide dismutase activities were significantly reduced in diabetic women; glutathione peroxidase was reduced in both those with gestational and type 1 diabetes, and superoxide dismutase was reduced only in type 1 diabetics [53]. 

### 2.5. Hypothalamic-Pituitary-Adrenal Axis

An increase in circulating glucocorticoids may play a role in early programming of adult disease. Experimental studies in animals and epidemiological studies in humans have suggested an altered set-point of the hypothalamic-pituitary-adrenal (HPA) axis as an important long-term change that occurs in association with reduced fetal growth. Pregnant animal models have shown that exposure to a variety of stressors, including nutrient restriction, results in the birth of offspring with elevated basal or stress-induced glucocorticoid secretion [54, 55]. It is thought that maternal exposure to stressors during pregnancy subsequently leads to excessive fetal exposure to glucocorticoids resulting in persistent alterations in HPA axis activity. In support of this hypothesis are studies conducted in rats in which fetoplacental exposure to maternally administered steroids throughout gestation reduced birth weight and produced hypertensive adult offspring. In one such study, dexamethasone administration to pregnant rats on days 15–20 of gestation resulted in offspring with reduced birth weight, elevated blood pressure, increased basal plasma corticosterone, lower mRNA expression of hippocampal neuronal glucocorticoid receptor, and decreased gene expression of hippocampal mineralocorticoid receptor [56]. In a sheep study, dams were treated with dexamethasone over 2 days; treatment group 1 was treated during 22–29 days of pregnancy and treatment group 2 was treated during 59–66 days of pregnancy (term 145 days) [57]. Offspring from dams that had received dexamethasone during 22–29 days gestation, but not days 59–66 of gestation, had elevated blood pressures. Such studies suggest that excess glucocorticoid exposure at certain developmental stages or “windows” programs higher blood pressure [57]. Another study of pregnant sheep found that severe brief undernutrition in late gestation altered the function of the HPA axis of adult offspring. Those exposed to gestational undernourishment for 10 days demonstrated altered steroid levels including an increased adrenocorticotropic hormone (ACTH) response as compared to offspring from dams fed *ad libitum* or offspring from dams undernourished for 20 days [58]. 

An epidemiological study in humans involving three populations found that adults who had lower birth weights had elevated fasting plasma concentrations of cortisol and that cortisol concentrations positively correlated with current blood pressure [59]. The authors concluded that increased activity of the HPA axis may link low birth weight with raised blood pressure in adult life [59]. In a study of over 300 men, plasma cortisol concentrations fell progressively with increasing birth weight; this trend was independent of age and body mass index [60]. Raised plasma cortisol concentrations were significantly associated with higher blood pressure as well as plasma glucose concentrations and insulin resistance, suggesting that programming of the HPA axis may be a mechanism underlying the association between low birth weight and the metabolic syndrome in adult life [60]. 

### 2.6. Neuropeptides

The hypothalamus plays a critical role in the regulation of appetite and body composition by way of responding to cues from neuropeptides. A series of studies, primarily in rodents, have explored the possibility that maternal nutrition during pregnancy may alter the level of energy intake in the offspring through inducing changes in the hypothalamic circuitry and in the expression, localization, and action of specific neuropeptides. Central and peripheral neuropeptides function in the regulation of appetite. Appetite stimulating neuropeptides include neuropeptide Y (NPY), agouti-related peptide (AgRP), and ghrelin. Conversely, appetite suppressing neuropeptides include cocaine and amphetamine-related transcript (CART), melanocyte-stimulating hormone (MSH), serotonin, insulin, and leptin [61, 62]. Of note is that the overweight and obese tend to develop resistance to insulin and leptin; therefore these two neuropeptides are usually elevated in such groups. The hypothalamus has multiple nerve centers, or nuclei, that are sensitive to a variety of physiologic changes. Hypothalamic nuclei include the arcuate nucleus (ARC), paraventricular nucleus (PVN), and ventromedial nucleus (VMN) which are involved in a number of biological activities with a fair degree of overlap [63]. In general, the ARC is a conduit of many diverse signals involved in various functions such as energy homeostasis [64], while the PVN is associated with blood pressure and stress responses [65] and the VMN with satiety [63]. Each also plays a role in the central nervous system regulation of food intake and body weight. 

Studies in rats have found that exposure to overnutrition in the fetal or neonatal period can result in permanent changes in body fat mass and in the hypothalamic neuronal circuitry regulating appetite in the adult brain. Dörner, Plagemann, and colleagues found immunocytochemical and morphometric aberrations in the hypothalamus of offspring of diabetic dams or in those exposed to milk from diabetic dams. In one of their early studies, offspring of diabetic dams had permanent hypoplasia of the VMN, decreased insulin responsiveness to glucose, impaired glucose tolerance, and increased susceptibility to diabetes [66]. A later study examined the effects of exposure to milk from diabetic dams. In this study, offspring of control dams cross-fostered to diabetic dams developed early postnatal growth delay and showed structural and functional hypothalamic “malprogramming” as it relates to appetite stimulation and suppression [67]. Exposure to milk from a diabetic dam resulted in an upregulation of the appetite stimulants NPY and AgRP and a downregulation of the appetite suppressant MSH. Although the researchers expected to find changes in the VMN as in their previous study, they found increased total number of neurons in the PVN which, as mentioned previously, is associated with regulation of blood pressure [67]. Incidentally, their research team performed a human, clinical study and reported an association between the neonatal ingestion of breast milk from diabetic mothers and increased blood pressure during childhood [68]. 

Leptin has received a considerable amount of attention in regard to early programming of appetite and body composition. In a rodent study to establish whether neonatal (postnatal day 3–13) leptin treatment can alleviate postnatal obesity and the associated metabolic sequelae that occur in the offspring of undernourished dams, leptin treatment reversed the programmed phenotype [69]. The benefits of leptin treatment in these rats included a slowing of neonatal weight gain and normalized caloric intake, body weight, fat mass, and fasting plasma glucose and insulin [69]. Studies in leptin deficient (Lep^ob^/Lep^ob^) mice have demonstrated permanently disrupted neural projection pathways from the ARC [70]. In the *adult *Lep^ob^/Lep^ob^ mice, leptin treatment did not reverse these neuroanatomical defects; however treatment of *neonatal *Lep^ob^/Lep^ob^ mice with exogenous leptin rescued the development of ARC projections [71]. 

### 2.7. Epigenetics

Epigenetics refers to all modifications to genes other than changes in the DNA sequence itself and includes DNA methylation and histone modifications [18, 72]. DNA methylation is a post-replication modification that is predominantly found in the cytosines of the dinucleotide sequence cytosine phosphate guanine (CpG) [73]. DNA methylation stabilizes gene expression in cells. Changes in methylation status (hyper- or hypomethylation) have been associated with various health conditions including malignancies [73]. Histones are proteins that permit the packaging of DNA into nucleosomes, the fundamental units of chromatin [74]. Histones are subject to a large number of post-translational modifications which are likely to control the structure and/or function of the chromatin fiber. Different modifications may have distinct consequences such as transcriptional activation or DNA repair [74]. 

Every cell in the body has the same genetic information; what makes cells, tissues, and organs different is that different sets of genes are turned on or expressed. An increasing body of evidence supports the role of environmentally-induced epigenetic changes in disease susceptibility. Experimental studies in agouti mice suggest a role for maternal diet in inducing epigenetic changes in the offspring [75, 76]. Agouti mice are so named because they carry the agouti gene [77]. Those carrying the dominant agouti alleles *A* ^*y*^ or *A* ^*vy*^ develop the complex set of traits collectively referred to as the yellow obese syndrome. The yellow obese syndrome encompasses the pleiotropic effects of yellow fur, obesity, hyperinsulinemia, hyperglycemia, and increased susceptibility to neoplasia [77]. Female *a/a* mice fed a methyl-supplemented diet with extra folate, vitamin B-12_,_ choline, and betaine 2 weeks prior to mating with male *A* ^*vy*^/*a* agouti mice and throughout pregnancy and lactation passed along the agouti gene to their offspring intact, yet demonstrated a shift in distribution towards having more offspring with the pseudoagouti phenotype [76]. Pseudoagouti mice are brown, lean, healthy, and longer lived than their yellow siblings [75]. This shift in the distribution in coat color was mediated by CpG methylation at the at the *A* ^*vy*^ locus [76]. Another example of environmentally-induced epigenetic changes is reduced pancreatic and duodenal homeobox 1 (Pdx1), also known as insulin promoter factor 1, in a rat model of intrauterine growth restriction. Pdx1 is a transcription factor necessary for development and function of the insulin producing pancreatic *β* cell. Rats faced with intrauterine growth restriction had permanently reduced expression of Pdx1 in *β* cells and developed type 2 diabetes in adulthood [78, 79]. Reduced Pdx1 transcription was mediated through a cascade of epigenetic modifications characterized by loss of upstream stimulatory factor-1 binding at the proximal promoter of Pdx1, recruitment of the histone deacetylase 1 and the corepressor Sin3A, and deacetylation of histones H3 and H4 which culminated in the eventual silencing of Pdx1 [78]. 

The Dutch famine of 1944 has been used by various investigators as an equivalent to an experimental study to investigate the effects of perinatal undernutrition in humans [80]. The ongoing Hunger Winter Families Study contributed empirical support for the hypothesis that early-life environmental conditions cause epigenetic changes in humans that persist throughout life. Individuals who had been exposed to famine during periconception had, 6 decades later, less DNA methylation of the insulin-like growth factor II (IGF2) gene compared with their unexposed, same-sex siblings [81]. The Dutch famine cohort has also been used to study the transgenerational effects of famine exposure. Women exposed to famine while in the womb later had offspring with birth weights lower than offspring of women not exposed to famine. This effect of *in utero* exposure to famine on birth weight in the subsequent generation persisted after control for potential confounding and intervening variables [82]. 

In a study of 2 prospective cohorts, Godfrey and colleagues used DNA extracted from umbilical cord tissue obtained at birth in children who were later assessed for adiposity at 9 years of age to measure methylation status of CpGs in the promoters of candidate genes [83]. Five candidate genes were selected based on a number of criteria which included animal data [83–85] and correlations with overall gene methylation status and adiposity. Methylation of retinoid X receptor-*α* (RXRA) chr9: 136355885+ (in cohort 1 and 2) and endothelial nitric oxide synthase (eNOS) chr7: 150315553+ (in cohort 1 only) at birth was correlated with greater adiposity in later childhood. Although the data is correlative and does not prove causality between DNA methylation at birth and adiposity in childhood, the observation suggests that epigenetics is involved in fetal programming of later obesity [83].

## 3. Macronutrient Contributions 

### 3.1. Protein

A sizable number of experimental studies, predominantly in rats, have explored the effects of protein restriction on fetal growth and later health. As discussed in the section on the thrifty phenotype hypothesis, protein restriction during pregnancy has been found to produce small offspring that have reduced numbers of nephrons which potentially contributes to the development of hypertension in adulthood [16, 34, 35]. Low protein exposure in fetal rats has also been shown to result in reduced pancreatic *β* cell mass at birth and reduced insulin secretion in later life presumably due to dietary-induced reduction in proliferation rate and increased apoptosis of *β* cells [86]. Weanling (26-day-old) rats exposed to low protein during gestation and lactation were smaller and had reduced serum insulin and increased serum glucose and triglycerides [87]. In addition, low protein-exposed offspring had increased hepatic triglycerides and hepatic expression of lipogenic enzymes favoring fat synthesis. The authors suggested that the increased expression of fat-synthesizing enzymes may account for the increased levels of serum and liver triglycerides seen in those exposed to low protein and that these increases may predispose the offspring to excessive accumulation of fat and ultimately obesity and insulin resistance [87]. Protein restriction may be involved in programming food preferences. Rat studies indicate that gestational exposure to low levels of protein due to maternal protein restriction establishes a preference for high fat foods [88]. When given the choice of selecting high fat, high protein, or high carbohydrate foods, 12-week-old offspring of dams fed a low protein diet throughout gestation consumed significantly more of the high fat food and significantly less of the high carbohydrate food than their control counterparts. At 30 weeks of age, there was no difference in the pattern of food selection between the two groups. Their results suggest that early exposure to undernutrition programs a preference for fatty foods, and thus maternal nutrition may influence pathways involved in control of appetite or the perception of palatability in the offspring [88]. 

Although the literature on protein restriction and disease risk is quite extensive, it is largely based on rodent models with few having assessed the specific role of protein restriction in human risk of disease. One study of men and women in Scotland sought to determine how diet of the mother in pregnancy influences blood pressure in the 40-year-old adult offspring [89]. The authors found that the relations between the diet of mothers and the blood pressure of their offspring were complex. When maternal intake of animal protein was less than 50 g daily, a higher carbohydrate intake was associated with higher blood pressures in the offspring. Conversely, when daily protein intake was above 50 g, lower carbohydrate intake was associated with higher blood pressure. An additional finding was that increases in blood pressure were associated with decreased placental size. Their conclusions were that blood pressure in adulthood may be influenced by maternal intakes of protein and carbohydrates during pregnancy and that this may be mediated through effects on placental growth [89]. 

### 3.2. Carbohydrates

All dietary carbohydrates can be converted into glucose [90]. In both animal and human studies, intrauterine exposure to high sugar diets and/or hyperglycemia has been found to increase the risk of the metabolic syndrome. In rats, a high fructose diet for 2 weeks resulted in increases in systolic blood pressure as well as plasma insulin and triglyceride concentrations as compared to rats fed a normal chow [91]. In another rat study early-and long-term exposure to a high sucrose diet (HSD) was assessed to determine whether such exposure alleviates the detrimental effects of sucrose feeding in later life [92]. Dams were fed either standard or HSD (70% calories as sucrose) starting 1 week before breeding and throughout gestation and lactation. After weaning, all male offspring were fed HSD until the age of 20 weeks, then detailed metabolic and morphometric profiles were ascertained. Offspring of sucrose-fed dams displayed higher adiposity and increases in triglyceride liver content together with higher low-density lipoprotein (LDL) cholesterol concentrations. Although the significance and mechanisms are not clear, a somewhat perplexing observation was that of substantial increases in the insulin sensitivity of skeletal muscle together with higher concentrations of adiponectin in the offspring of sucrose-fed dams compared with the offspring of standard diet-fed dams. Triglycerides, free fatty acids, overall glucose tolerance, and the insulin sensitivity of adipose tissue were comparable in both groups [92]. 

Data derived from the Camden Study of adolescent pregnancy implicated high sugar consumption with a twofold increased risk for delivering small for gestational age infants [93]. Additionally, evaluation of the data based on ethnicity led to the detection of a substantial decrease in the duration of gestation among Puerto Rican adolescents with high sugar diets, whereas there was no effect of a high sugar diet on gestation duration among black and white adolescents. The authors commented that other studies involving comparisons among ethnicities have found Hispanic women to be more likely to have abnormalities of carbohydrate metabolism during pregnancy as evidenced by abnormal glucose tolerance tests and increased incidence of gestational diabetes [93–95]. Pregnancies complicated by maternal diabetes, in any form, place the offspring at risk for developing obesity and glucose intolerance [29, 96]. In a study of 18- to 27-year-old women born to diabetic (gestational diabetes or type 2 diabetes) mothers, the risk of overweight was doubled in the offspring of diabetic mothers as compared with offspring from a background population [97]. Moreover, the risk of the metabolic syndrome was increased 4-fold for offspring of mothers with gestational diabetes and 2.5-fold for offspring of mothers with type 1 diabetes. Offspring risk of the metabolic syndrome increased significantly with increasing maternal fasting blood glucose as well as 2-hour blood glucose following an oral glucose load. Their findings indicate that intrauterine exposure to hyperglycemia contributes to the pathogenesis of the metabolic syndrome, thus offspring of diabetic mothers are risk groups for this condition [97]. 

### 3.3. Fats

Our laboratory has performed multiple experimental studies aimed at determining the role of high fat diet in maternal-fetal health. In one such study, mice exposed to a high saturated fat diet during gestation and lactation developed adult obesity, hyperglycemia, insulin resistance, and hypertension, despite being fed a standard rodent diet post-weaning [98]. Given that oxidative stress is a potential mechanism connecting fetal exposures to the development of the metabolic syndrome, a group of pregnant mice were supplemented with quercetin, a powerful antioxidant, which mitigated the detrimental effects of high fat diet exposure [98]. Similarly, Bouanane and colleagues used a rat model to determine the effects of a cafeteria diet, that had 42% of energy from fat, on oxidant/antioxidant status as well as variety of metabolic markers [99]. Dams fed the cafeteria diet developed increased body weight, hyperglycemia, hyperinsulinemia, hyperleptinemia, hyperlipidemia, and an imbalance between oxidants/antioxidants favoring oxidative stress. Male and female offspring displayed similar effects, which persisted after birth, suggesting an unrelenting effect of maternal diet on metabolism and body habitus of surviving offspring [99]. In the development of a dietary-induced gestational diabetes mouse model, our laboratory demonstrated that consumption of a high saturated fat diet prior to conception and throughout pregnancy can result in insulin resistance and placental vascular damage and that these abnormalities could be a result of oxidative stress [100]. Frias and colleagues, using a nonhuman primate model to determine the effect of chronic high fat diet on pregnancy outcome, found that consumption of such a diet, independent of maternal obesity, led to a significant reduction in uterine blood flow, a rise in placental inflammation, and an increase in fetal risk of nonalcoholic fatty liver disease, as evidenced by increased levels of liver triglycerides and increased hepatic oxidative stress [101, 102]. Offspring of high fat diet dams also exhibited elevated hepatic expression of gluconeogenic enzymes and transcription factors. Moreover, reversing the maternal high fat diet to a low fat diet during a subsequent pregnancy improved fetal hepatic triglyceride levels and partially normalized gluconeogenic enzyme expression. The authors concluded that a developing fetus is highly vulnerable to excess lipids, independent of maternal diabetes and/or obesity and that such exposure may increase the risk of pediatric fatty liver disease [102]. 

Human observational studies have also found high fat intakes to be related to pregnancy and birth outcome as well as long-term health of the offspring. In a case-control study of 912 women admitted to obstetric hospitals for spontaneous abortion, the risk of miscarriage was directly associated with consumption of the main types of fats, butter and oil, while a significant inverse relationship was established between the risk of spontaneous abortion and the consumption of green vegetables, fruits, milk, cheese, eggs, and fish [103]. Results of a comprehensive dietary review of women with a history of gestational diabetes found that women with recurrence of gestational diabetes in a subsequent pregnancy had significantly higher fat intakes as a percentage of total energy than women who did not have recurrence [104]. Lipids and lipid disorders play a central role in the development of the metabolic syndrome and its associated diseases [105]. Atherosclerotic lesions are known to begin in early life [106]. Fatty streaks in the aorta have been observed in children as young as 3 years old [107] and autopsies of young soldiers killed in the Korean and Vietnam wars revealed advanced coronary artery lesions [108, 109]. Similarly, the Pathobiological Determinants of Atherosclerosis in Youth (PDAY) study investigated atherosclerosis in autopsied persons 15 to 34 years old that died from various traumas and observed advanced lesions of atherosclerosis in adolescents and young adults and that serum lipoprotein concentrations were a risk factor [110]. 

## 4. Micronutrient Contributions 

 Although micronutrient deficiencies are known causes of several well-characterized diseases, such as scurvy and rickets [111], the role of micronutrients in the fetal origins of adult disease has yet to be explored in detail. However, existing evidence points to their potential importance [112]. Many micronutrients are antioxidants or are components of the antioxidant defense system. The section on oxidative stress provided examples on how micronutrients, as antioxidants, may be associated with various short- and long-term effects on the offspring. In relating maternal micronutrient intake with fetal glucocorticoid exposure, a mouse model of maternal dietary restriction of copper, zinc, and vitamin E demonstrated reduced activity level of placental 11*β*-hydroxysteroid dehydrogenase-2, an enzyme that protects the fetus from overexposure to maternal glucocorticoids [113]. As mentioned in the section on the hypothalamic-pituitary-adrenal axis, fetal exposure to glucocorticoids is associated with small size at birth and insulin resistance and hypertension in adult life. Indeed, in this study offspring exposed to the micronutrient restricted diet had significantly reduced body weight and crown-to-rump length at birth as well as increased systolic blood pressure and insulin levels post-weaning as compared to those exposed to a control diet. As discussed in the section on epigenetics, experimental studies on the impact of methyl-supplemented diets containing added folate and vitamin B-12 revealed the potential role of these micronutrients in inducing major changes in offspring phenotype, including the ability to impact the development of chronic diseases. Studies on maternal dietary zinc restriction in rats indicate that zinc deficiency during intrauterine and postnatal growth can induce elevations in blood pressure and renal lesions in adulthood [114, 115]. Concerning the renal alterations identified, early exposure to zinc deficiency resulted in a decrease in glomerular filtration rate which was associated with a reduction in the number and size of nephrons. These animals also had proteinuria, higher lipid peroxidation end products, and evidence of increased renal apoptosis and fibrosis [114, 115]. 

Few human studies have explored the role of early micronutrient deficiencies in the development of adult metabolic disorders. Of the existing reports, results are often conflicting and the significance is ambiguous. In two randomized controlled trials, both in Nepal, one found that daily multiple micronutrient supplementation in pregnant women resulted in slightly lower blood pressure in the offspring at 2.5 years of age [116], while the other found no effects on blood pressure with maternal multiple micronutrient supplementation among 6- to 8-year-old offspring [117]. As part of the Pune (India) Maternal Nutrition Study, higher maternal erythrocyte folate concentrations at 28 weeks gestation were associated with higher adiposity and insulin resistance in children 6 years of age [118]. Conversely, a study in England found that maternal folate intake at 18 or 32 weeks gestation was not associated with any measures of body composition in children at 9 years of age [119]. Clearly more work is needed to disentangle the intricate interactions among micronutrients and to determine how these interactions may be involved in the initiation and progression of chronic disease. 

## 5. Conclusions

 It is now widely accepted that certain chronic diseases of adulthood may have their origins in the womb. Studies discussed in this paper provide evidence that a mother's diet during pregnancy can exert major effects on the short- and long-term health of her children including programming of the metabolic syndrome. The challenges at present are to identify common mechanisms and pathways involved in disparate perinatal malnutrition paradigms, deciphering physiological and/or pathological roles of specific nutrients, and to determine which components of the maternal diet may be best modified to optimize maternal health, placental integrity, birth outcome, and lifelong health of the offspring. The implications of this avenue of research, particularly to obstetric and preventative medicine, dictate that effective interdisciplinary communication and knowledge transfer occur and that the information generated is disseminated to the general public. A recent review provides encouraging evidence that prenatal nutritional advice appears effective in reducing the risk of preterm birth and that balanced energy and protein supplementation seem to improve fetal growth and may reduce the risk of stillbirth and infants born small for gestational age [120]. 

Whilst the evidence in support of the DOHaD is compelling, there are many caveats and controversies in the field including species differences, body weight verses body composition, and twin studies. First, species differences (e.g., maternal age, parity, gestation length, fetal number, and variability in fetoplacental development) should be considered when results derived from animal studies are extrapolated to humans. Rodents are particularly important to reproductive and obstetric studies because of the strong similarities between human and murine placentas [121–123]; however, there are many differences between rodents and humans particularly relating to early development. Rodents are altricial species, and their organs mature after birth, which explains why postnatal factors may be more pertinent in rodent species, whereas pre- and perinatal factors play a vital role in human development. Human infants of hyperglycemic mothers develop macrosomia, whereas hyperglycemic rodent dams typically have normally sized or small pups. The more precocious human *β* cells produce fetal insulin prior to birth, thus increasing glucose transport and growth prenatally, which does not occur in the rodent, whose endocrine pancreas becomes functional postnatally [62, 124, 125]. Second, studies relating early exposures to later life risk of disease have been largely based on body weight and/or body mass index; however, these measurements provide no information on body composition. More recent studies have included the assessment of body composition, and evidence suggests that lower birth weights reflect increased relative adiposity, while higher birth weights reflect higher lean mass [15]. The increased use of body composition analysis is likely to offer a more complete understanding as to why small babies are prone to the development of the metabolic syndrome, an association that likely involves increased adiposity and decreased lean mass at birth. Finally, studies in twins, especially monozygotic twins, have caused considerable contention in the field [15, 29]. Twins face both physical and nutrient constraints and are generally smaller than singletons. The fetal origins hypothesis indicates that such individuals should have increased morbidity and mortality from metabolic and cardiovascular disease, but this has not been proven to be necessarily the case [15, 29, 126]. The DOHaD is a relatively new field of research and in time such discrepancies may be resolved. With a more complete understanding of the role of maternal health and nutrition in the initiation and progression of the metabolic syndrome and other disorders comes the hope of prevention of chronic diseases at their earliest beginnings. 

## References

[1] Ford ES, Giles WH, Dietz WH (2002). Prevalence of the metabolic syndrome among US adults: findings from the Third National Health and Nutrition Examination Survey. *Journal of the American Medical Association*.

[2] Alberti KGMM, Zimmet P, Shaw J (2006). Metabolic syndrome: a new world-wide definition. A consensus statement from the International Diabetes Federation. *Diabetic Medicine*.

[3] Eckel RH, Grundy SM, Zimmet PZ (2005). The metabolic syndrome. *The Lancet*.

[4] Forsdahl A (1977). Are poor living conditions in childhood and adolescence an important risk factor for arteriosclerotic heart disease?. *British Journal of Preventive and Social Medicine*.

[5] Barker DJ, Osmond C (1988). Low birth weight and hypertension. *British Medical Journal*.

[6] Barker DJP, Winter PD, Osmond C, Margetts B, Simmonds SJ (1989). Weight in infancy and death from ischaemic heart disease. *The Lancet*.

[7] Hales CN, Barker DJP, Clark PMS (1991). Fetal and infant growth and impaired glucose tolerance at age 64. *British Medical Journal*.

[8] Barker DJP, Hales CN, Fall CHD, Osmond C, Phipps K, Clark PMS (1993). Type 2 (non-insulin-dependent) diabetes mellitus, hypertension and hyperlipidaemia (syndrome X): relation to reduced fetal growth. *Diabetologia*.

[9] Ozanne SE, Hales CN (2002). Early programming of glucose-insulin metabolism. *Trends in Endocrinology and Metabolism*.

[10] Vangen S, Nordhagen R, Lie KK (2005). Revisiting the Forsdahl-Barker hypothesis. *Journal of the Norwegian Medical Association*.

[11] Jehn M, Brewis A (2009). Paradoxical malnutrition in mother-child pairs: untangling the phenomenon of over- and under-nutrition in underdeveloped economies. *Economics and Human Biology*.

[12] Black RE, Allen LH, Bhutta ZA (2008). Maternal and child undernutrition: global and regional exposures and health consequences. *The Lancet*.

[13] Kelly T, Yang W, Chen CS, Reynolds K, He J (2008). Global burden of obesity in 2005 and projections to 2030. *International Journal of Obesity*.

[14] Sobal J, Stunkard AJ (1989). Socioeconomic status and obesity: a review of the literature. *Psychological Bulletin*.

[15] Rogers I (2003). The influence of birthweight and intrauterine environment on adiposity and fat distribution in later life. *International Journal of Obesity and Related Metabolic Disorders*.

[16] Gluckman PD, Hanson MA (2004). The developmental origins of the metabolic syndrome. *Trends in Endocrinology and Metabolism*.

[17] Barker DJP (2007). The origins of the developmental origins theory. *Journal of Internal Medicine*.

[18] Cota BM, Allen PJ (2010). The developmental origins of health and disease hypothesis. *Pediatric Nursing*.

[19] Gluckman PD, Hanson MA, Pinal C (2005). The developmental origins of adult disease. *Maternal and Child Nutrition*.

[20] Lee TM, Zucker I (1988). Vole infant development is influenced perinatally by maternal photoperiodic history. *The American Journal of Physiology*.

[21] Lee TM, Spears N, Tuthill CR, Zucker I (1989). Maternal melatonin treatment influences rates of neonatal development of meadow vole pups. *Biology of Reproduction*.

[22] Barraclough CA (1961). Production of anovulatory, sterile rats by single injections of testosterone propionate. *Endocrinology*.

[23] Harding JE (2001). The nutritional basis of the fetal origins of adult disease. *International Journal of Epidemiology*.

[24] Bateson P, Barker D, Clutton-Brock T (2004). Developmental plasticity and human health. *Nature*.

[25] Kanaka-Gantenbein C (2010). Fetal origins of adult diabetes. *Annals of the New York Academy of Sciences*.

[26] Bavdekar A, Yajnik CS, Fall CHD (1999). Insulin resistance syndrome in 8-year-old Indian children: small at birth, big at 8 years, or both?. *Diabetes*.

[27] Norris SA, Osmond C, Gigante D (2012). Size at birth, weight gain in infancy and childhood, and adult diabetes risk in five low- or middle-income country birth cohorts. *Diabetes Care*.

[28] Lear SA, Kohli S, Bondy GP, Tchernof A, Sniderman AD (2009). Ethnic variation in fat and lean body mass and the association with insulin resistance. *Journal of Clinical Endocrinology and Metabolism*.

[29] McMillen IC, Robinson JS (2005). Developmental origins of the metabolic syndrome: prediction, plasticity, and programming. *Physiological Reviews*.

[30] Hales CN, Barker DJP (1992). Type 2 (non-insulin-dependent) diabetes mellitus: the thrifty phenotype hypothesis. *Diabetologia*.

[31] Hofman PL, Cutfield WS, Robinson EM (1997). Insulin resistance in short children with intrauterine growth retardation. *Journal of Clinical Endocrinology and Metabolism*.

[32] Simmons RA, Gounis AS, Bangalore SA, Ogata ES (1992). Intrauterine growth retardation: fetal glucose transport is diminished in lung but spared in brain. *Pediatric Research*.

[33] Simmons RA, Flozak AS, Ogata ES (1993). The effect of insulin and insulin-like growth factor-I on glucose transport in normal and small for gestational age fetal rats. *Endocrinology*.

[34] Mackenzie HS, Brenner BM (1995). Fewer nephrons at birth: a missing link in the etiology of essential hypertension?. *The American Journal of Kidney Diseases*.

[35] Hinchliffe SA, Lynch MRJ, Sargent PH, Howard CV, Van Velzen D (1992). The effect of intrauterine growth retardation on the development of renal nephrons. *British Journal of Obstetrics and Gynaecology*.

[36] Haycock GB (1998). Development of glomerular filtration and tubular sodium reabsorption in the human fetus and newborn. *British Journal of Urology*.

[37] Manalich R, Reyes L, Herrera M, Melendi C, Fundora I (2000). Relationship between weight at birth and the number and size of renal glomeruli in humans: a histomorphometric study. *Kidney International*.

[38] Ong KKL, Ahmed ML, Dunger DB, Emmett PM, Preece MA (2000). Association between postnatal catch-up growth and obesity in childhood: prospective cohort study. *British Medical Journal*.

[39] Cianfarani S, Germani D, Branca F (1999). Low birthweight and adult insulin resistance: the “catch-up growth” hypothesis. *Archives of Disease in Childhood*.

[40] Luo ZC (2006). Tracing the origins of “fetal origins” of adult diseases: programming by oxidative stress?. *Medical Hypotheses*.

[41] Bhargava SK, Sachdev HS, Fall CHD (2004). Relation of serial changes in childhood body-mass index to impaired glucose tolerance in young adulthood. *The New England Journal of Medicine*.

[42] Leger J, Noel M, Limal JM, Czernichow P (1996). Growth factors and intrauterine growth retardation. II. Serum growth hormone, insulin-like growth factor (IGF) I, and IGF-binding protein 3 levels in children with intrauterine growth retardation compared with normal control subjects: prospective study from birth to two years of age. *Pediatric Research*.

[43] Cianfarani S, Germani D, Rossi P (1998). Intrauterine growth retardation: evidence for the activation of the insulin-like growth factor (IGF)-related growth-promoting machinery and the presence of a cation-independent IGF binding protein-3 proteolytic activity by two months of life. *Pediatric Research*.

[44] Law CM, Shiell AW, Newsome CA (2002). Fetal, infant, and childhood growth and adult blood pressure: a longitudinal study from birth to 22 years of age. *Circulation*.

[45] Adair LS, Martorell R, Stein AD (2009). Size at birth, weight gain in infancy and childhood, and adult blood pressure in 5 low- and middle-income-country cohorts: when does weight gain matter?. *The American Journal of Clinical Nutrition*.

[46] Barker DJP, Osmond C, Forsén TJ, Kajantie E, Eriksson JG (2005). Trajectories of growth among children who have coronary events as adults. *The New England Journal of Medicine*.

[47] Singhal A, Fewtrell M, Cole TJ, Lucas A (2003). Low nutrient intake and early growth for later insulin resistance in adolescents born preterm. *The Lancet*.

[48] Willcox JK, Ash SL, Catignani GL (2004). Antioxidants and prevention of chronic disease. *Critical Reviews in Food Science and Nutrition*.

[49] Lenzen S (2008). Oxidative stress: the vulnerable *β*-cell. *Biochemical Society Transactions*.

[50] Ceriello A, Motz E (2004). Is oxidative stress the pathogenic mechanism underlying insulin resistance, diabetes, and cardiovascular disease? The common soil hypothesis revisited. *Arteriosclerosis, Thrombosis, and Vascular Biology*.

[51] Gupta P, Narang M, Banerjee BD, Basu S (2004). Oxidative stress in term small for gestational age neonates born to undernourished mothers: a case control study. *BMC Pediatrics*.

[52] Baydas G, Karatas F, Gursu MF (2002). Antioxidant vitamin levels in term and preterm infants and their relation to maternal vitamin status. *Archives of Medical Research*.

[53] Peuchant E, Brun JL, Rigalleau V (2004). Oxidative and antioxidative status in pregnant women with either gestational or type 1 diabetes. *Clinical Biochemistry*.

[54] Lingas R, Dean F, Matthews SG (1999). Maternal nutrient restriction (48 h) modifies brain corticosteroid receptor expression and endocrine function in the fetal guinea pig. *Brain Research*.

[55] Phillips DI (2001). Fetal growth and programming of the hypothalamic-pituitary-adrenal axis. *Clinical and Experimental Pharmacology and Physiology*.

[56] Levitt NS, Lindsay RS, Holmes MC, Seckl JR (1996). Dexamethasone in the last week of pregnancy attenuates hippocampal glucocorticoid receptor gene expression and elevates blood pressure in the adult offspring in the rat. *Neuroendocrinology*.

[57] Dodic M, May CN, Wintour EM, Coghlan JP (1998). An early prenatal exposure to excess glucocorticoid leads to hypertensive offspring in sheep. *Clinical Science*.

[58] Bloomfield FH, Oliver MH, Giannoulias CD, Gluckman PD, Harding JE, Challis JRG (2003). Brief undernutrition in late-gestation sheep programs the hypothalamic-pituitary-adrenal axis in adult offspring. *Endocrinology*.

[59] Phillips DIW, Walker BR, Reynolds RM (2000). Low birth weight predicts elevated plasma cortisol concentrations in adults from 3 populations. *Hypertension*.

[60] Phillips DIW, Barker DJP, Fall CHD (1998). Elevated plasma cortisol concentrations: a link between low birth weight and the insulin resistance syndrome?. *Journal of Clinical Endocrinology and Metabolism*.

[61] Arora S, Anubhuti (2006). Role of neuropeptides in appetite regulation and obesity: a review. *Neuropeptides*.

[62] McMillen IC, Adam CL, Mühlhäusler BS (2005). Early origins of obesity: programming the appetite regulatory system. *Journal of Physiology*.

[63] Elmquist JK, Elias CF, Saper CB (1999). From lesions to leptin: hypothalamic control of food intake and body weight. *Neuron*.

[64] Cone RD, Cowley MA, Butler AA, Fan W, Marks DL, Law MJ (2001). The arcuate nucleus as a conduit for diverse signals relevant to energy homeostasis. *International Journal of Obesity*.

[65] Guyenet PG (2006). The sympathetic control of blood pressure. *Nature Reviews Neuroscience*.

[66] Dörner G, Plagemann A, Ruckert J (1988). Teratogenetic maternofoetal transmission and prevention of diabetes susceptibility. *Experimental and Clinical Endocrinology and Diabetes*.

[67] Fahrenkrog S, Harder T, Stolaczyk E (2004). Cross-fostering to diabetic rat dams affects early development of mediobasal hypothalamic nuclei regulating food intake, body weight, and metabolism. *Journal of Nutrition*.

[68] Harder T, Franke K, Plagemann A, Kohlhoff R (2001). Early nutrition and later blood pressure: effect of maternal diabetes. *Journal of Pediatrics*.

[69] Vickers MH, Gluckman PD, Coveny AH (2005). Neonatal leptin treatment reverses developmental programming. *Endocrinology*.

[70] Bouret SG, Draper SJ, Simerly RB (2004). Formation of projection pathways from the arcuate nucleus of the hypothalamus to hypothalamic regions implicated in the neural control of feeding behavior in mice. *Journal of Neuroscience*.

[71] Bouret SG, Draper SJ, Simerly RB (2004). Trophic action of leptin on hypothalamic neurons that regulate feeding. *Science*.

[72] Wolffe AP, Matzke MA (1999). Epigenetics: regulation through repression. *Science*.

[73] Jaenisch R, Bird A (2003). Epigenetic regulation of gene expression: how the genome integrates intrinsic and environmental signals. *Nature Genetics*.

[74] Peterson CL, Laniel MA (2004). Histones and histone modifications. *Current Biology*.

[75] Wolff GL, Kodell RL, Moore SR, Cooney CA (1998). Maternal epigenetics and methyl supplements affect agouti gene expression in A^(vy)^/a mice. *Journal of the Federation of American Societies for Experimental Biology*.

[76] Waterland RA, Jirtle RL (2003). Transposable elements: targets for early nutritional effects on epigenetic gene regulation. *Molecular and Cellular Biology*.

[77] Miltenberger RJ, Mynatt RL, Wilkinson JE, Woychik RP (1997). The role of the agouti gene in the yellow obese syndrome. *Journal of Nutrition*.

[78] Park JH, Stoffers DA, Nicholls RD, Simmons RA (2008). Development of type 2 diabetes following intrauterine growth retardation in rats is associated with progressive epigenetic silencing of Pdx1. *Journal of Clinical Investigation*.

[79] Simmons RA, Templeton LJ, Gertz SJ (2001). Intrauterine growth retardation leads to the development of type 2 diabetes in the rat. *Diabetes*.

[80] Roseboom TJ (2011). Hungry in the womb: what are the consequences? Lessons from the Dutch famine. *Maturitas*.

[81] Heijmans BT, Tobi EW, Stein AD (2008). Persistent epigenetic differences associated with prenatal exposure to famine in humans. *Proceedings of the National Academy of Sciences of the United States of America*.

[82] Lumey LH (1992). Decreased birthweights in infants after maternal in utero exposure to the Dutch famine of 1944-1945. *Paediatric and Perinatal Epidemiology*.

[83] Godfrey KM, Sheppard A, Gluckman PD (2011). Epigenetic gene promoter methylation at birth is associated with child’s later adiposity. *Diabetes*.

[84] Burdge GC, Lillycrop KA (2010). Nutrition, epigenetics, and developmental plasticity: implications for understanding human disease. *Annual Review of Nutrition*.

[85] Lillycrop KA, Rodford J, Garratt ES (2010). Maternal protein restriction with or without folic acid supplementation during pregnancy alters the hepatic transcriptome in adult male rats. *British Journal of Nutrition*.

[86] Petrik J, Reusens B, Arany E (1999). A low protein diet alters the balance of islet cell replication and apoptosis in the fetal and neonatal rat and is associated with a reduced pancreatic expression of insulin-like growth factor-II. *Endocrinology*.

[87] Maloney CA, Gosby AK, Phuyal JL, Denyer GS, Bryson JM, Caterson ID (2003). Site-specific changes in the expression of fat-partitioning genes in weanling rats exposed to a low-protein diet in utero. *Obesity Research*.

[88] Bellinger L, Lilley C, Langley-Evans SC (2004). Prenatal exposure to a maternal low-protein diet programmes a preference for high-fat foods in the young adult rat. *British Journal of Nutrition*.

[89] Campbell DM, Hall MH, Barker DJP, Cross J, Shiell AW, Godfrey KM (1996). Diet in pregnancy and the offspring’s blood pressure 40 years later. *British Journal of Obstetrics and Gynaecology*.

[90] Ludwig DS (2002). The glycemic index: physiological mechanisms relating to obesity, diabetes, and cardiovascular disease. *Journal of the American Medical Association*.

[91] Hwang I, Ho H, Hoffman BB, Reaven GM (1987). Fructose-induced insulin resistance and hypertension in rats. *Hypertension*.

[92] Šedová L, Šeda O, Kazdová L (2007). Sucrose feeding during pregnancy and lactation elicits distinct metabolic response in offspring of an inbred genetic model of metabolic syndrome. *The American Journal of Physiology*.

[93] Lenders CM, Hediger ML, Scholl TO, Khoo CS, Slap GB, Stallings VA (1997). Gestational age and infant size at birth are associated with dietary sugar intake among pregnant adolescents. *Journal of Nutrition*.

[94] Berkowitz GS, Lapinski RH, Wein R, Lee D (1992). Race/ethnicity and other risk factors for gestational diabetes. *The American Journal of Epidemiology*.

[95] Green JR, Pawson IG, Schumacher LB, Perry J, Kretchmer N (1990). Glucose tolerance in pregnancy: ethnic variation and influence of body habitus. *The American Journal of Obstetrics and Gynecology*.

[96] Dörner G, Plagemann A (1994). Perinatal hyperinsulinism as possible predisposing factor for diabetes mellitus, obesity and enhanced cardiovascular risk in later life. *Hormone and Metabolic Research*.

[97] Clausen TD, Mathiesen ER, Hansen T (2009). Overweight and the metabolic syndrome in adult offspring of women with diet-treated gestational diabetes mellitus or type 1 diabetes. *Journal of Clinical Endocrinology and Metabolism*.

[98] Liang C, Oest ME, Prater MR (2009). Intrauterine exposure to high saturated fat diet elevates risk of adult-onset chronic diseases in C57BL/6 mice. *Birth Defects Research B*.

[99] Bouanane S, Benkalfat NB, Baba Ahmed FZ (2009). Time course of changes in serum oxidant/antioxidant status in overfed obese rats and their offspring. *Clinical Science*.

[100] Liang C, DeCourcy K, Prater MR (2010). High-saturated-fat diet induces gestational diabetes and placental vasculopathy in C57BL/6 mice. *Metabolism*.

[101] Frias AE, Morgan TK, Evans AE (2011). Maternal high-fat diet disturbs uteroplacental hemodynamics and increases the frequency of stillbirth in a nonhuman primate model of excess nutrition. *Endocrinology*.

[102] McCurdy CE, Bishop JM, Williams SM (2009). Maternal high-fat diet triggers lipotoxicity in the fetal livers of nonhuman primates. *Journal of Clinical Investigation*.

[103] Di Cintio E, Parazzini F, Chatenoud L (2001). Dietary factors and risk of spontaneous abortion. *European Journal of Obstetrics Gynecology and Reproductive Biology*.

[104] Moses RG, Shand JL, Tapsell LC (1997). The recurrence of gestational diabetes: could dietary differences in fat intake be an explanation?. *Diabetes Care*.

[105] Grundy SM (1998). Hypertriglyceridemia, atherogenic dyslipidemia, and the metabolic syndrome. *The American Journal of Cardiology*.

[106] Berenson GS, Wattigney WA, Tracy RE (1992). Atherosclerosis of the aorta and coronary arteries and cardiovascular risk factors in persons aged 6 to 30 years and studied at necropsy (The Bogalusa Heart Study). *The American Journal of Cardiology*.

[107] Holman RL, McGill HC, Strong JP, Geer JC (1958). The natural history of atherosclerosis: the early aortic lesions as seen in New Orleans in the middle of the of the 20th century. *The American journal of pathology*.

[108] Enos WF, Holmes RH, Beyer J (1986). Coronary disease among United States soldiers killed in action in Korea: preliminary report. *Journal of the American Medical Association*.

[109] McNamara JJ, Molot MA, Stremple JF, Cutting RT (1971). Coronary artery disease in combat casualties in Vietnam. *Journal of the American Medical Association*.

[110] Strong JP (1995). Natural history and risk factors for early human atherogenesis. Pathobiological Determinants of Atherosclerosis in Youth (PDAY) Research Group. *Clinical Chemistry*.

[111] Goldsmith L, Katz S, Gilchrest B, Paller A, Leffell D, Wolff K (2012). Micronutrients. *Fitzpatrick'S Dermatology in General Medicine*.

[112] Christian P, Stewart CP (2010). Maternal micronutrient deficiency, fetal development, and the risk of chronic disease. *Journal of Nutrition*.

[113] Rosario JF, Gomez MP, Anbu P (2008). Does the maternal micronutrient deficiency (copper or zinc or vitamin E) modulate the expression of placental 11 *β* hydroxysteroid dehydrogenase-2 per se predispose offspring to insulin resistance and hypertension in later life?. *Indian Journal of Physiology and Pharmacology*.

[114] Tomat AL, Inserra F, Veiras L (2008). Moderate zinc restriction during fetal and postnatal growth of rats: effects on adult arterial blood pressure and kidney. *The American Journal of Physiology*.

[115] Tomat AL, Costa MDLT, Arranz CT (2011). Zinc restriction during different periods of life: influence in renal and cardiovascular diseases. *Nutrition*.

[116] Vaidya A, Saville N, Shrestha BP, de L Costello AM, Manandhar DS, Osrin D (2008). Effects of antenatal multiple micronutrient supplementation on children’s weight and size at 2 years of age in nepal: follow-up of a double-blind randomised controlled trial. *The Lancet*.

[117] Stewart CP, Christian P, Schulze KJ, LeClerq SC, West KP, Khatry SK (2009). Antenatal micronutrient supplementation reduces metabolic syndrome in 6- to 8-year-old children in rural nepal. *Journal of Nutrition*.

[118] Yajnik CS, Deshpande SS, Jackson AA (2008). Vitamin B12 and folate concentrations during pregnancy and insulin resistance in the offspring: the Pune Maternal Nutrition Study. *Diabetologia*.

[119] Lewis SJ, Leary S, Davey Smith G, Ness A (2009). Body composition at age 9 years, maternal folate intake during pregnancy and methyltetrahydrofolate reductase (MTHFR) C677T genotype. *British Journal of Nutrition*.

[120] Ota E, Tobe-Gai R, Mori R, Farrar D (2012). Antenatal dietary advice and supplementation to increase energy and protein intake. *Cochrane Database of Systematic Reviews*.

[121] Georgiades P, Fergyson-Smith AC, Burton GJ (2002). Comparative developmental anatomy of the murine and human definitive placentae. *Placenta*.

[122] Cox B, Kotlyar M, Evangelou AI (2009). Comparative systems biology of human and mouse as a tool to guide the modeling of human placental pathology. *Molecular Systems Biology*.

[123] Carter AM (2007). Animal models of human placentation: a review. *Placenta*.

[124] Elahi MM, Cagampang FR, Mukhtar D, Anthony FW, Ohri SK, Hanson MA (2009). Long-term maternal high-fat feeding from weaning through pregnancy and lactation predisposes offspring to hypertension, raised plasma lipids and fatty liver in mice. *British Journal of Nutrition*.

[125] Symonds ME, Sebert SP, Budge H (2009). The impact of diet during early life and its contribution to later disease: critical checkpoints in development and their long-term consequences for metabolic health. *Proceedings of the Nutrition Society*.

[126] Vågerö D, Leon D (1994). Ischaemic heart disease and low birth weight: a test of the fetal-origins hypothesis from the Swedish twin registry. *The Lancet*.

